# Nail changes in pemphigus and bullous pemphigoid: A single-center study in China

**DOI:** 10.3389/fmed.2022.933608

**Published:** 2022-09-20

**Authors:** Shan Cao, Xiaochen Cui, Jianke Li, Futang Pan, Xiaoxiao Yan, Qing Yang, Mingfei Chen, Shengji Zhou, Donghong Du, Weiwei Wang, Yuanhang Sun, Zhongxiang Shi, Mei Wu, Baoqi Yang, Furen Zhang

**Affiliations:** Department of Dermatology, Shandong Provincial Hospital for Skin Diseases & Shandong Provincial Institute of Dermatology and Venereology, Shandong First Medical University & Shandong Academy of Medical Sciences, Jinan, China

**Keywords:** autoimmune bullous diseases, pemphigus, bullous pemphigoid, nail, disease severity, antibodies

## Abstract

Common autoimmune bullous diseases (AIBDs) include pemphigus and bullous pemphigoid (BP), which are primarily caused by IgG autoantibodies against the structural proteins of desmosomes at the cell–cell junction and hemidesmosomes at the epidermal–dermal junction. Few studies have assessed nail changes in patients with pemphigus or BP. In the present study, we collected the clinical data of 191 patients with AIBDs (108 patients with pemphigus and 83 patients with BP) and 200 control subjects. Nail changes were observed in 77.0% (147/191), 77.8% (84/108), and 75.9% (63/83) of patients with AIBDs, pemphigus, and BP, respectively, and 14.5% (29/200) of control subjects. Beau's lines and paronychia were the most common nail involvement, observed in 22.5% (43/191) and 22.5% (43/191) of patients with AIBDs, 25.0% (27/108) and 25.9% (28/108) of patients with pemphigus, 19.3% (16/83) and 18.1% (15/83) of patients with BP, respectively. The autoimmune bullous skin disorder intensity score (ABSIS) and the onset time of patients with pemphigus or BP with nail changes were different. Onychomycosis accounted for 21.5% (41/191) of all patients with AIBDs. The ABSIS was correlated with nail involvement in patients with BP (*r* = 0.46, *p* < 0.001), and weakly correlated with nail involvement in patients with AIBDs (*r* = 0.37, *p* < 0.001), pemphigus (*r* = 0.29, *p* = 0.009), and pemphigus vulgaris (PV; *r* = 0.35, *p* = 0.008). No correlation was observed between nail involvement and disease antibody titers. In conclusion, nail changes are frequently observed in patients with pemphigus and BP. The type and onset time of nail changes may indicate the severity of pemphigus and BP, which warrants the attention of dermatologists.

## Introduction

Autoimmune bullous diseases (AIBDs) are a heterogeneous group of diseases characterized by the development of cutaneous and mucosal vesicles, blisters, and erosions. Pemphigus and bullous pemphigoid (BP) represent the two major types of AIBDs caused by circulating autoantibodies against the desmosomal antigens desmoglein (Dsg) 1 and Dsg3 and hemidesmosomal antigens BP180 and BP230, respectively. Pemphigus vulgaris (PV) and pemphigus foliaceus (PF) are the most common forms of pemphigus (1). These dermatoses are characterized by the production of pathogenic autoantibodies that react with desmosomal proteins or hemidesmosomal antigens (2).

Autoimmune bullous diseases may cause nail changes (i.e., Beau's lines and paronychia) when target antigens are expressed in the hyponychium, nail matrix, and proximal nail fold, except for skin and mucosal lesions (3, 4). The incidence of nail involvement in patients with AIBDs is different in different studies (4–6). Previous studies have reported nail involvement in 13.4–47.0% of patients with PV (6, 7) and 71.4% of patients with BP (8). One study suggested that hemorrhagic nail abnormalities might be associated with a poor prognosis in patients with PV (9). The above findings indicate that nail involvement may be a clinical sign correlated with the severity of pemphigus and BP (4, 8–12).

In this study, we aimed to investigate the clinical characteristics of nail changes and their relationships with disease severity in Chinese patients with pemphigus and BP.

## Methods

We retrospectively analyzed the clinical nail data of patients diagnosed with pemphigus or BP at Shandong Provincial Hospital for Skin Diseases from January 2019 to December 2020. The diagnosis of pemphigus and BP was made on the basis of clinical manifestations and the results of histological and immunological examinations [i.e., direct immunofluorescence, indirect immunofluorescence, and enzyme-linked immunosorbent assay (ELISA)]. Age- and sex-matched healthy subjects were recruited as control subjects. The study was conducted according to the guidelines of the Declaration of Helsinki, and all patients provided written informed consent. This study was approved by the Ethics Committee of the Shandong Provincial Hospital for Skin Diseases (Approval no. 20181225KYKT024).

Nail characteristics were classified according to each type of nail change (4, 8, 11, 13). Disease severity was evaluated by the autoimmune bullous skin disorder intensity score (ABSIS) and antibody titers. The ABSIS, ranging from 0 to 206, was obtained by scoring the quality of skin lesions, indicating both disease activity and damage (14). The score of nail changes was calculated based on the type and location of nail changes: one type of nail change = 1 point; one location of nail change = 1 point. The total score is the sum of the scores of nail change types and nail change locations. Spearman's correlation coefficient (*r*) was used for the correlation analysis. A *p* < 0.05 was considered statistically significant.

## Results

**Figure 1:**
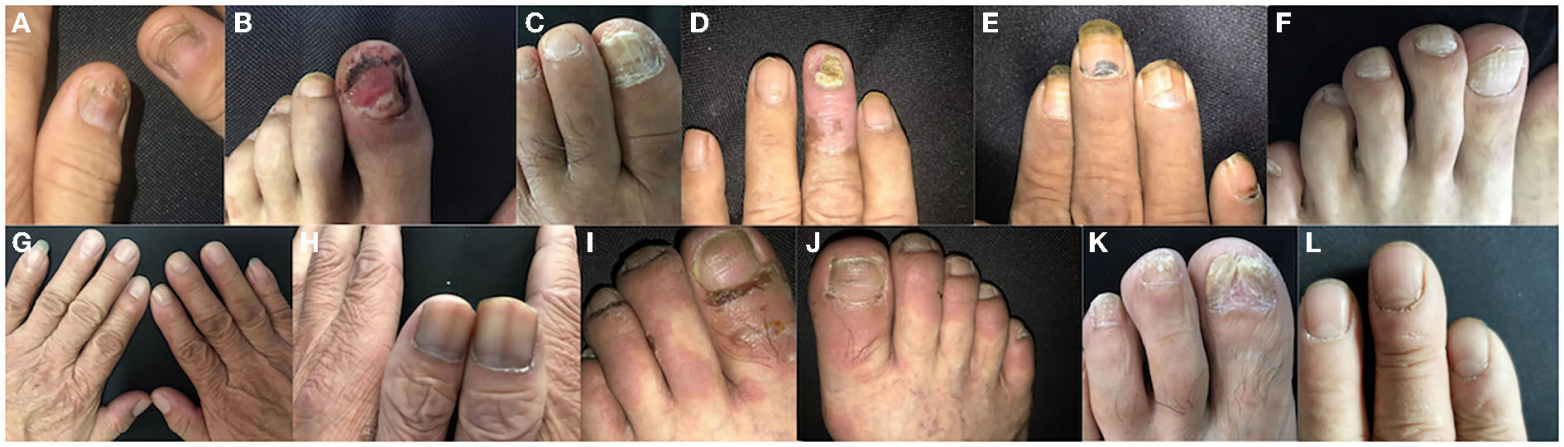
Various types of nail involvement in patients with autoimmune bullous disease (AIBDs). **(A)** Beau's lines, **(B)** paronychia, **(C)** onycholysis, **(D)** onychomycosis, **(E)** subungual hemorrhage, **(F)** onychorrhexis, **(G)** longitudinal ridging, **(H)** nail discoloration, **(I)** periungual bullae, **(J)** nail pitting, **(K)** pterygium, and **(L)** Muehrcke's lines (transverse leukonychia).

A total of 191 patients with AIBDs, including 108 patients with pemphigus and 83 patients with BP, and 200 control subjects were collected in this study. Patients with pemphigus and BP had a mean age of 56.5 ± 9.2 and 64.8 ± 12.2 years, respectively. Patients with pemphigus and BP had a male to female ratio of 1.3:1 and 1.5:1, respectively. Overall, 77.8% (84/108) of patients with pemphigus and 75.9% (63/83) of patients with BP exhibited nail involvement, compared to 14.5% (29/200) of control subjects (Table 1). The duration between disease onset and nail changes varied between 0.5–12.8 and 2.0–6.6 months, respectively. Nail involvement occurred in 77.0% (147/191) of patients with AIBDs (Figure 1). Among them, 96.6% (142/147) had nail changes after the onset of skin or mucosal lesions.

**Table 1:** General characteristics of patients with pemphigus or bullous pemphigoid.

**Types**	**Number**	**Age (Mean±SE)**	**Male-to-female**	**Number of patients with nail changes (%)**	**ABSIS (Mean±SE)**
PV	68	54.5 ± 14.0	1.1:1	57 (83.8)	69.9 ± 37.2
PF	40	54.5 ± 12.5	1.4:1	27 (67.5)	66.6 ± 39.3
Pemphigus	108	56.5 ± 9.2	1.3:1	84 (77.8)	67.3 ± 39.1
BP	83	64.8 ± 12.2	1.5:1	63 (75.9)	56.5 ± 24.4
AIBDs	191	65.4 ± 12.4	1.4:1	147 (77.0)	63.0 ± 33.9
Controls	200	52.5 ± 6.2	1.3:1	29 (14.5)	none

ABSIS, autoimmune bullous skin disorder intensity score; PV, pemphigus vulgaris; PF, pemphigus foliaceus; BP, bullous pemphigoid; AIBDs, autoimmune bullous diseases, including pemphigus and BP.

Beau's lines [22.5% (43/192)] and paronychia [22.5% (43/192)] were the most common types of nail changes in patients with AIBDs, followed by onycholysis [21.5% (41/191)]. Beau's lines, paronychia, and onychomycosis were observed in 0, 0, and 7.0% (14/200) of control subjects, respectively. Paronychia was the most common nail involvement in patients with pemphigus [25.9% (28/108)]. Onychomycosis was observed in 22.2% (24/108) of patients with pemphigus and 20.5% (17/83) of patients with BP, which was higher than the control group [7.0% (14/200)]. Pterygium was observed in one patient with BP. Muehrcke's lines (transverse leukonychia) were observed in one patient with relapsed PV. However, no nail changes were observed at the first onset of pemphigus. All other types of nail involvement occurred in patients with pemphigus and BP (Table 2). The highest ABSIS was observed in patients with onychomycosis, which accounted for 21.5% (41/191) of all patients with AIBDs. Various types and onset time of nail changes with different ABSIS of patients with pemphigus and BP. Paronychia, Beau's lines, subungual hemorrhage, and onycholysis of the nails usually represent a higher ABSIS.

**Table 2:** Clinical characteristics and frequency of nail involvement in patients with pemphigus or bullous pemphigoid.

**Nail changes**	**AIBDs cases (%)**	**Pemphigus cases (%)**	**BP cases (%)**	**Controls (%)**	**AIBDs ABSIS (Mean±SE)**	**Onset days (Mean±SE)**
Beau's lines	43 (22.5)	27 (24.8)	16 (19.3)	0	68.4 ± 38.6	53.2 ± 54.2
Paronychia	43 (22.5)	28 (25.7)	15 (18.1)	0	73.6 ± 40.1	52.5 ± 68.8
Onycholysis	41 (21.5)	27 (24.8)	14 (16.9)	5 (2.5)	62.6 ± 31.2	57.33 ± 70.71
Onychorrhexis	33 (17.3)	18 (16.5)	15 (18.1)	6 (3.0)	64.7 ± 37.2	78.1 ± 72.4
Subungual hemorrhage	32 (16.8)	23 (21.1)	9 (10.8)	1 (0.5)	71.2 ± 38.6	46.8 ± 49.2
Longitudinal ridging	30 (15.7)	21 (19.3)	9 (10.8)	1 (0.5)	66.0 ± 37.3	65.9 ± 81.1
Nail discoloration	21 (11.0)	9 (8.3)	12 (14.5)	2 (1.0)	57.9 ± 22.4	54.3 ± 36.9
Perungual bullae	11 (5.8)	4 (3.7)	7 (8.4)	0	55.0 ± 27.7	89.9 ± 96.3
Nail pitting	3 (1.6)	1 (0.9)	2 (2.4)	0	55.7 ± 22.8	110.0 ± 113.6
Pterygium	1 (0.5)	0	1 (1.2)	0	32.0	none
Muehrcke's lines	1 (0.5)	1 (0.9)	0	0	66.0	none

ABSIS, autoimmune bullous skin disorder intensity score; PV, pemphigus vulgaris; PF, pemphigus foliaceus; BP, bullous pemphigoid.

The score of nail changes, ranging between 0 and 25, showed mild to severe nail changes in patients with pemphigus and BP. One patient with PV had the highest score of nail changes. This patient's fingernails and toenails showed different degrees of nail changes with Beau's lines, paronychia, onycholysis, subungual hemorrhage, and onychorrhexis.

Nail changes were correlated with the ABSIS in patients with BP (*r* = 0.46, *p* < 0.001). There was a weak correlation between ABSIS and the nail involvement score in patients with AIBDs (*r* = 0.37, *p* < 0.001), pemphigus (*r* = 0.29, *p* = 0.009), and PV (*r* = 0.35, *p* = 0.008), and no correlation in patients with PF (*r* = 0.14, *p* = 0.48). There was no significant correlation between nail involvement and anti-DSG1/DSG3 antibody titers in patients with pemphigus (*r* = 0.14, *p* = 0.22*; r* = 0.09, *p* = 0.44, respectively), PV (*r* = 0.16, *p* = 0.24; *r* = 0.17, *p* = 0.23, respectively), or PF (*r* = 0.07, *p* = 0.73; *r* = −0.128, *p* = 0.53, respectively). There was also no significant correlation between nail involvement and anti-BP180/BP230 antibody titers in patients with BP (*r* = 0.18, *p* = 0.16; *r* = −0.10, *p* = 0.44, respectively) (Table 3).

**Table 3:** Spearman's correlation coefficient (r) of disease severity with nail changes in patients with pemphigus or bullous pemphigoid.

**AIBDs subtype**	**ABSIS**	**Anti-Dsg1/BP180 antibody**	**Anti-Dsg3/BP230 antibody**
PV	*r* = 0.35, *P* = 0.008	*r* = 0.16, *P* = 0.24	*r* = 0.17, *P* = 0.23
PF	*r* = 0.14, *P* = 0.48	*r* = 0.07, *P* = 0.73	*r* = −0.128, *P* = 0.53
Pemphigus	*r* = 0.29, *P* <0.001	*r* = 0.14, *P* = 0.22	*r* = 0.09, *P* = 0.44
BP	*r* = 0.46, *P* <0.001	*r* = 0.18, *P* = 0.16	*r* = −0.10, *P* = 0.44
AIBDs	*r* = 0.37, *P* <0.001	*r* = 0.17, *P* = 0.05	*r* = 0.06, *P* = 0.46

AIBDs, autoimmune bullous disease; ABSIS, autoimmune bullous skin disorder intensity score; Dsg, desmoglein; BP, bullous pemphigoid; PV, pemphigus vulgaris; PF, pemphigus foliaceus.

## Discussion

Nail changes may be associated with skin lesions as an AIBDs diathesis (5, 8–11, 15, 16). The incidence of nail involvement in patients with PV was 47.0% and 31.6% in two retrospective studies (*n* = 64 and *n* = 79, respectively) (7, 12). A case-control study in India showed a significant association between the severity of AIBDs (29 cases with pemphigus and seven cases with BP) and nail changes (*p* = 0.0021) (8). Recently, a retrospective study of 448 patients with PV and 41 patients with PF found that fingernail changes were common in PV and were associated with the ABSIS (10). Future studies need to validate these findings, which summarize the clinical characteristics of different types of nails and their relationships with disease severity, especially in patients with BP.

Nail involvement in AIBDs occurs either before or in conjunction with a flare of pre-existing disease, and is rarely a part of the initial presentation (8, 11, 12). It is consistent with our observation that 96.6% (142/147) of nail involvement in patients with AIBDs occurs after the onset of cutaneous or mucosae lesions.

In patients with pemphigus, paronychia was associated with a higher ABSIS than either Beau's lines or onycholysis, consistent with previous findings (10). Patients with paronychia had the second highest ABSIS, either at initial presentation or during disease exacerbation, with the prevalence of pemphigus. AIBD-associated paronychia may be distinguished from other similar clinical presentations of acute or chronic paronychia and bacterial infections (4), which are considered a sign of exacerbation and can be cured by systemic therapy, nor with topical therapy (6, 16–18). The prevalence of Beau's lines or onychomadesis (nail shedding) may be related to the formation of proximal nail fold and nail matrix, which are temporarily blocked by antigens to reduce the formation of nail board proteins (4, 8). Onychomycosis was observed in 22.2% (24/108) of patients with pemphigus, with an increased prevalence among patients undergoing immunosuppressive therapy. This is consistent with previous studies (4, 7, 18). Patients with pemphigus with nail pitting showed the longest onset than those with other types of nail changes, but had a moderate ABSIS. This data suggest that nail pitting may take a long time to develop and cause a mild damage to the nail. Nail pitting, periungual bullae, and nail discoloration occurred in patients with PV and BP, which was consistent with the findings that patients with PV and BP had a higher ABSIS than those with PF.

Muehrcke's lines (transverse leukonychia) occurred in one patient with PV when he relapsed. However, no nail changes were observed at the first onset. The ABSIS of the patient at relapse was higher than that at the first onset. Muehrcke's lines are usually associated with chronic hypoalbuminemia secondary to other diseases (13). However, our patient did not have hypoalbuminemia. Muehrcke's lines induce edema in the connective tissues in front of the lunula, just below the epidermis of the nail bed, altering the compact arrangement of the collagen in this area to one with a looser texture coinciding with hypoalbuminemia (13). The structure resembled the lunula, indicating a relationship with the severity of PV.

Onychomycosis is found in 20.5% (17/83) of patients with BP, probably because onychomycosis occurs following secondary nail changes, accompanied by paronychia, Beau's lines, onycholysis, onychorrhexis, nail discoloration, longitudinal ridging, subungual hemorrhage, and nail pitting, or as a result of immunosuppressive therapy (4, 7, 19). In our study, most patients with pemphigus or BP were treated with immunosuppressive therapy. Pterygium was found in only one patient with BP, with a relatively low ABSIS score. Fingernail pterygium has been reported in a few patients with cicatricial pemphigoid and paraneoplastic pemphigus (4, 8). Here, one patient developed pterygium when BP relapsed. We assumed that pterygium might be associated with chronic recurrent inflammatory conditions. Patients with subungual hemorrhage had the shortest onset time and higher ABSIS among all pemphigus and BP cases, suggesting that this type of nail change occurs rapidly and represents a more severe condition. Subungual hemorrhage may indicate overall disease severity in most cases of pemphigus and BP. A higher prevalence of onychorrhexis was observed in previous studies (30%) (8) than in our study (17.3%).

Generally occurring or specific nail changes (i.e., paronychia, Beau's lines, subungual hemorrhage, and onycholysis) in patients with pemphigus and BP are caused by the pathology and not by drug cytotoxicity (11). The proximal nail fold, nail matrix, and hyponychium express all basement membrane zone (BMZ) antigens and components (4, 20). The type of nail changes may indicate the disease severity, onset time, and location of the nail involvement according to the pemphigus or BP antigen damage (4). Nail changes are determined by the location of blistering in the nail apparatus, and may include onychomadesis, paronychia, and pterygium (4).

The limitation of this study is that most of the patients are inpatients with a high level of disease severity and a high ABSIS. Also, we found no significant correlation between the different types of nail changes and pemphigus/BP antibody titers. These findings will be validated by future studies with a larger sample size.

In conclusion, the different types and onset times of nail changes represent different severities of pemphigus and BP. Paronychia, Beau's lines, subungual hemorrhage, and onycholysis are associated with a higher ABSIS score, which warrants the attention of dermatologists. The mechanisms of nail changes in pemphigus and BP require further investigation.

## Data availability statement

The raw data supporting the conclusions of this article will be made available by the authors, without undue reservation.

## Ethics statement

The studies involving human participants were reviewed and approved by Ethics Committee of the Shandong Provincial Hospital for Skin Diseases. Written informed consent to participate in this study was provided by the participants' legal guardian/next of kin. Written informed consent was obtained from the individual(s) for the publication of any potentially identifiable images or data included in this article.

## Author contributions

SC and BY contributed to the conception, design of the study, wrote the manuscript, analyzed, and interpreted the data set. SC, XC, JL, FP, XY, QY, MC, SZ, DD, WW, YS, ZS, MW, BY, and FZ organized the database. All authors contributed to manuscript revision, read, and approved the submitted version.

## Conflict of interest

The authors declare that the research was conducted in the absence of any commercial or financial relationships that could be construed as a potential conflict of interest.

## Publisher's note

All claims expressed in this article are solely those of the authors and do not necessarily represent those of their affiliated organizations, or those of the publisher, the editors and the reviewers. Any product that may be evaluated in this article, or claim that may be made by its manufacturer, is not guaranteed or endorsed by the publisher.
